# Propofol prevents further prolongation of QT interval during liver transplantation

**DOI:** 10.1038/s41598-022-08592-4

**Published:** 2022-03-17

**Authors:** Seung Hyun Kim, Jae Geun Lee, Hyang Mi Ju, SuYoun Choi, Hyukjin Yang, Bon-Nyeo Koo

**Affiliations:** 1 Department of Anesthesiology and Pain Medicine, Anesthesia and Pain Research Institute, Severance Hospital, Yonsei University College of Medicine, 50-1 Yonsei-ro, Seodaemun-gu, Seoul, 120-752 Republic of Korea; 2 Department of Surgery, Yonsei University College of Medicine, Seoul, Republic of Korea; 3 Department of Anesthesiology and Pain Medicine, Yongin Severance Hospital, Yonsei University College of Medicine, Seoul, Republic of Korea; 4 Department of Anesthesiology and Pain Medicine, Severance Hospital, Yonsei University College of Medicine, Seoul, Republic of Korea

**Keywords:** Randomized controlled trials, Hepatology

## Abstract

Here, we aimed to compare the effects of two anesthetic methods (desflurane inhalation anesthesia vs. propofol-based total intravenous anesthesia (TIVA)] on corrected QT interval (QTc) values during living donor liver transplantation. Altogether, 120 patients who underwent living donor liver transplantation were randomized to either the desflurane or TIVA group. The primary outcome was intraoperative QTc change. Other electrocardiogram, hemodynamic findings and postoperative outcomes were examined as secondary outcomes. QTc values were prolonged intraoperatively in both groups; however, the change was smaller in the TIVA group than in the desflurane group (P_Group × Time_ < 0.001). More patients had QTc values of > 500 ms in the desflurane group than in the TIVA group (63.3% vs. 28.3%, *P* < 0.001). In patients with preoperative QTc prolongation, QTc was further prolonged in the desflurane group, but not in the TIVA group (P_Group × Time_ < 0.001). Intraoperative norepinephrine and vasopressin use were higher in the desflurane group than in the TIVA group. Propofol-based TIVA may reduce QTc prolongation during living donor liver transplantation compared to that observed with desflurane inhalational anesthesia, particularly in patients with preoperative QTc prolongation. Additionally, patients managed with propofol-based TIVA required less vasopressor during the procedure as compared with those managed with desflurane inhalational anesthesia.

## Introduction

Cirrhotic cardiomyopathy is characterized by ventricular hypertrophy, diastolic dysfunction, hyperdynamic circulation, and repolarization disorders, including prolonged QT interval^1^. Prolongation of the QT interval may cause ventricular arrhythmias such as torsades de pointes and sudden cardiac death^2,3^, which may occur during liver transplantation^4–6^.

Heart rate affects the QT interval, which is longer in bradycardia and shorter in tachycardia; thus, heart rate-corrected QT interval (QTc) is used clinically. Concurrently, the Tp-e interval, which is the interval between the peak and end of the T wave, may indicate the degree of ventricular repolarization^7^; combined with the Tp-e/QTc ratio, it may be used as an indicator of ventricular repolarization abnormality^8–11^.

Desflurane inhalational anesthesia is commonly used during liver transplantation; however, desflurane is associated with the prolongation of QTc^12–14^. Meanwhile, the effect of total intravenous anesthesia (TIVA) using propofol on QTc prolongation remains controversial^15^. Propofol has been used to prevent QTc prolongation, and, in some cases, to shorten the QTc interval^16–19^. However, QTc prolongation induced by propofol has also been reported^20,21^. No previous study has compared the effect of desflurane and TIVA using propofol on QTc prolongation during liver transplantation. This study aimed to evaluate the effect of these anesthetic methods on the QTc interval, Tp-e interval, and Tp-e/QT values during liver transplantation.

## Results

**Figure 1:**
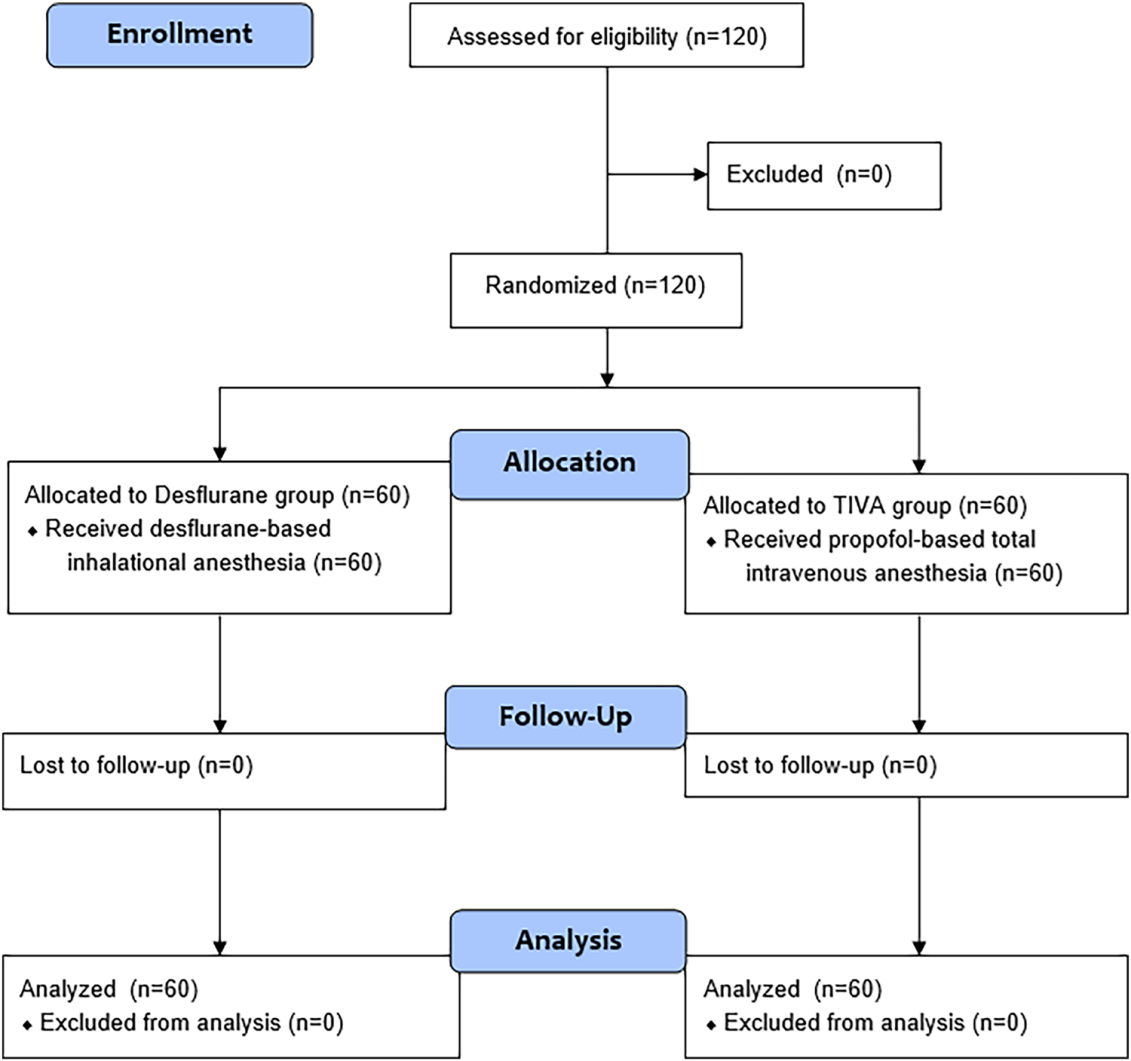
CONSORT flowchart, capturing the study design.

A total of 120 patients were randomly allocated to the desflurane or TIVA group. Figure 1 shows the CONSORT flow diagram for this study. Baseline patient characteristics including age, sex, body mass index, American Society of Anesthesiologists’ physical status classification, model for end-stage liver disease score, disease etiology (alcohol abuse or non-alcohol abuse), proportion of patients with preoperative QTc prolongation and rates of preoperative comorbidities, as well as preoperative complications related to liver cirrhosis were similar in both groups (Table 1). Preoperative cardiac medications, including beta blockers (propranolol and carvedilol) and calcium channel blocker (amlodipine) were also comparable between the two groups. Among the patients with beta blocker use, six patients in the desflurane group and five in the TIVA group maintained their medication until the morning of surgery. Three out of four patients with calcium channel blocker use in the TIVA group maintained their medication until the morning of surgery. Antibiotic use was also similar between the two groups. Between both groups, a total of 90 patients were administered 1 g of ceftazidime three times a day, while 26 patients were administered 4 g of piperacillin and 0.5 g of tazobactam three times a day. The remaining four patients were administered 0.5 g of meropenem twice a day.

**Table 1:** Baseline patient characteristics.

	Desflurane group (n = 60)	TIVA group (n = 60)	*P* value
Age (years)	58.0 (49.3, 63.0)	58.0 (53.0, 63.8)	0.351
Sex (Male/Female)	38/22	42/18	0.439
BMI (kg/m^2^)	23.7 (21.0, 26.3)	23.4 (21.9, 25.5)	0.410
ASA (3/4)	33/27	31/29	0.714
MELD score (score)	12.0 (8.0, 17.4)	10.5 (7.1, 15.0)	0.141
Etiology (nonalcoholics/alcoholics)	35/25	44/16	0.083
Preoperative QTc prolongation (n, %)	20 (33.3)	14 (23.3)	0.224
**Preoperative comorbidity**			
Hepatocellular carcinoma (n, %)	29 (48.3)	36 (32.5)	0.200
HTN (n, %)	12 (20.0)	15 (25.0)	0.512
DM (n, %)	27 (45.0)	19 (31.7)	0.133
Lung disease (n, %)	4 (6.7)	2 (3.3)	0.679
Heart disease (n, %)	6 (10.0)	3 (5.0)	0.491
CRF (n, %)	4 (6.7)	3 (5.0)	> 0.999
Pulmonary HTN (n, %)	3 (5.0)	2 (3.3)	> 0.999
**Preoperative complications**			
Ascites (n, %)	35 (58.3)	26 (43.3)	0.100
Hepatic encephalopathy (n, %)	17 (28.3)	10 (16.7)	0.126
Esophageal varix (n, %)	33 (55.0)	32 (53.3)	0.855
**Preoperative medication**			
Beta blocker (n, %)	10 (16.7)	8 (13.3)	0.609
Propranolol (n, %)	7 (11.7)	4 (6.7)	0.343
Carvedilol (n, %)	3 (5.0)	4 (6.7%)	> 0.999
Calcium channel blocker (n, %)	0 (0.0)	4 (6.7)	0.119
**Perioperative antibiotics**			> 0.999
Ceftazidime (n, %)	45 (75.0)	45 (75.0)	
Piperacillin + tazobactam (n, %)	13 (21.7)	13 (21.7)	
Meropenem (n, %)	2 (3.3)	2 (3.3)	

Data are presented as median (interquartile range) for continuous variables and count (percentage) for categorical variables.

**Figure 2:**
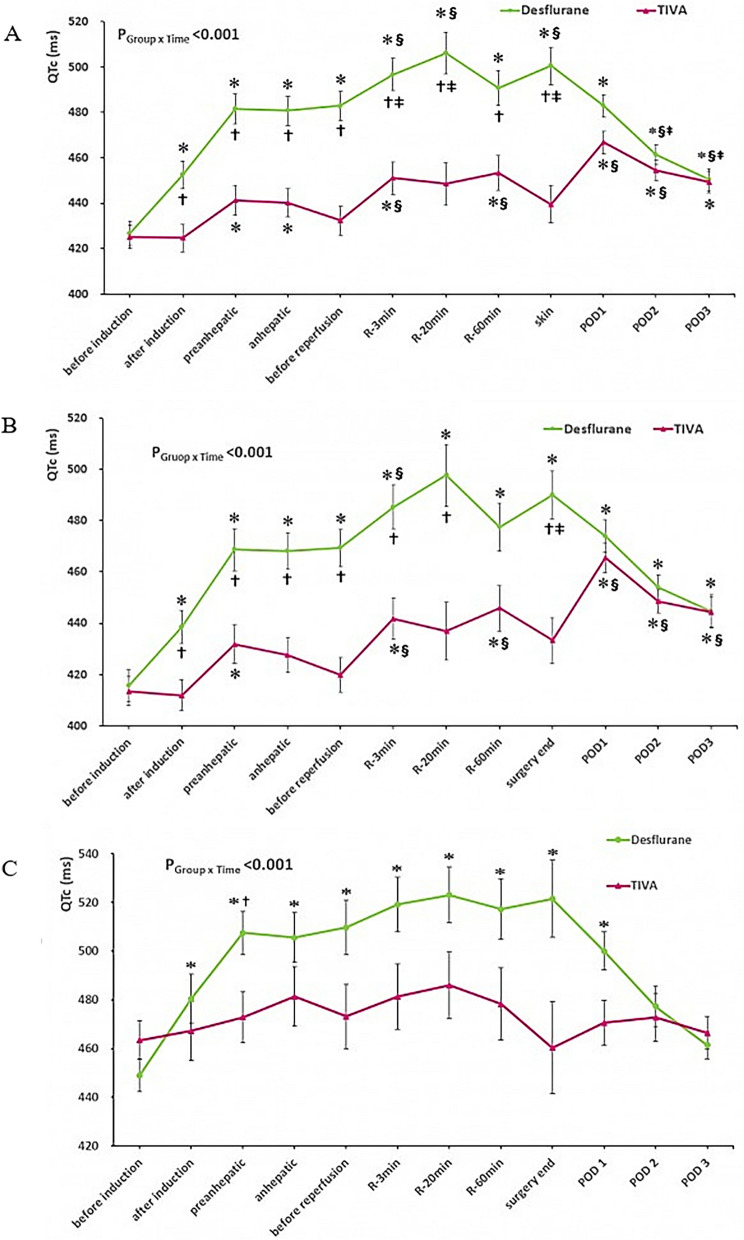
Intraoperative changes in heart rate corrected QT (QTc) intervals calculated by Bazett’s formula. Values are mean ± standard error. (**A**) QTc changes in all patients, (**B**) QTc changes in patients with normal preoperative QTc, (**C**) QTc changes in patients with preoperative QTc prolongation. *Bonferroni-corrected *P* values of < 0.05, compared with the value obtained before induction within the groups. §Bonferroni-corrected *P* values of < 0.05, compared with the value obtained before reperfusion within the groups. †Bonferroni-corrected *P* values of < 0.05, compared with the value obtained before induction between the groups. ‡Bonferroni-corrected *P* values of < 0.05 compared with the value of before reperfusion between the groups.

QTc were prolonged intraoperatively in both groups; however, these changes differed between the groups (P_Group × Time_ < 0.001; Fig. 2A); specifically, the prolongation was greater (relative to baseline) in the desflurane group than in the TIVA group throughout surgery (P_Group × Time_ < 0.001). Relative to values observed at 5 min before reperfusion, at 3 and 20 min after surgery and at the end of surgery, changes to QTc were greater in the desflurane group than in the TIVA group (P_Group × Time_ < 0.001). At the end of surgery, QTc values were prolonged in the desflurane group, but returned to baseline in the TIVA group. However, in both groups, postoperative QTc values were higher than the baseline values assessed in the operating room until postoperative day 3. Relative to the preoperative QTc values assessed in the ward, postoperative QTc values were prolonged until postoperative days 1 and 2, returning to preoperative level on postoperative day 3.

Two patients in the TIVA group and no patient in the desflurane group showed severely prolonged baseline QTc of > 500 ms; meanwhile, the proportion of patients with maximum QTc level of > 500 ms during surgery was higher in the desflurane group than in the TIVA group (38/60 [63.3%] vs. 17/60 [28.3%], *P* < 0.001). The Tp-e interval increased intraoperatively in both groups; however, changes to this parameter were similar in both groups (P_Group × Time_ = 0.1886; Table 2). The Tp-e/QTc ratio did not increase during surgery in either group.

**Table 2:** Changes in Tpeak-Tend (Tp-e) interval and Tp-e/QT ratio during surgery.

	Desflurane group (n = 60)	TIVA group (n = 60)	P_time*group_
**Tp-e interval (ms)**			0.1886
Before induction	57.8 (1.4)	57.4 (1.4)	
After induction	59.9 (1.7)	59.7 (1.7)	
Preanhepatic	61.2 (1.8)	58.5 (1.8)	
Anhepatic	62.2 (1.9) a	56.9 (1.9)	
5 min before reperfusion	62.9 (1.7) a	57.8 (1.7)	
3 min after reperfusion	66.2 (1.9) a,b	62.9 (1.9) a,b	
20 min after reperfusion	65.5 (2.1) a	61.8 (2.1) a,b	
60 min after reperfusion	68.4 (3.3) a	61.8 (3.3)	
Surgery end	68.4 (2.8) a,b	65.3 (2.8) a,b	
**Tp-e/QT ratio**			0.0590
Before induction	0.14 (0.0)	0.14 (0.0)	
After induction	0.13 (0.0)	0.14 (0.0)	
Preanhepatic	0.13 (0.0) a	0.13 (0.0)	
Anhepatic	0.13 (0.0)	0.13 (0.0)	
5 min before reperfusion	0.13 (0.0)	0.13 (0.0)	
3 min after reperfusion	0.13 (0.0)	0.14 (0.0)	
20 min after reperfusion	0.13 (0.0)	0.16 (0.0)	
60 min after reperfusion	0.14 (0.0)	0.14 (0.0)	
Surgery end	0.14 (0.0)	0.15 (0.0) a,b	

Data are presented as estimated mean (standard error).

TIVA, total intravenous anesthesia.

a Bonferroni-corrected *P* < 0.05 compared with the value of before induction in each group.

b Bonferroni-corrected *P* < 0.05 compared with the value of 5 min before reperfusion in each group.

c Bonferroni-corrected *P* < 0.05 compared with the TIVA group.

### Subgroup analysis (preoperative normal vs. prolonged QTc group)

Among patients with normal preoperative QTc, the QTc were prolonged intraoperatively in both groups; the observed changes differed between the groups (P_Group × Time_ < 0.001; Fig. 2B). In contrast, in patients with preoperative QTc prolongation, intraoperative QTc was prolonged only in the desflurane group (P_Group × Time_ < 0.001; Fig. 2C); this prolongation (relative to baseline) was maintained until postoperative day 1.

### Intraoperative hemodynamic data

The mean BP decreased after reperfusion in both groups; there was no intergroup difference in the mean BP change. Heart rate increase during surgery was higher in the desflurane group than in the TIVA group (P_Group × Time_ = 0.004; Table 3). While the cardiac index and stroke volume index values increased, systemic vascular resistance values decreased during surgery. The right ventricle end diastolic volume index values decreased during the preanhepatic and anhepatic phases, but not after reperfusion. Changes to hemodynamic index values, including right ventricle end diastolic volume index, systemic vascular resistance, and mixed venous oxygen saturation values were similar in both groups (Table 3). However, changes to cardiac index and stroke volume index values were greater in the desflurane group than in the TIVA group at 3 min and 20 min after reperfusion. Body temperatures at each timepoint did not differ significantly between the two groups (Supplementary Table S1).

**Table 3:** Intraoperative hemodynamic data.

	Desflurane group (n = 60)	TIVA group (n = 60)	P_time*group_
**Mean BP (mmHg)**			0.2675
Before induction	87.4 (1.8)	90.8 (1.8)	
After induction	82.1 (2.4)	81.3 (2.4) a	
Preanhepatic	85.1 (2.2)	92.9 (2.2)	
Anhepatic	86.6 (1.7)	86.7 (1.7)	
5 min before reperfusion	87.8 (2.0)	89.1 (2.0)	
3 min after reperfusion	90.7 (2.4)	89.5 (2.4)	
20 min after reperfusion	75.7 (1.5) a,b	77.7 (1.5) a,b	
60 min after reperfusion	76.3 (1.3) a,b	79.2 (1.3) a,b	
Surgery end	78.5 (1.4) a,b	82.7 (1.4) a,b	
**Heart rate (/min)**			0.0004
Before induction	79.2 (1.9)	79.0 (1.9)	
After induction	80.9 (2.0)	74.6 (2.0)	
Preanhepatic	86.3 (1.7) a	78.0 (1.7)	
Anhepatic	92.3 (2.2) a	92.1 (2.2) a	
5 min before reperfusion	86.6 (1.9) a	87.7 (1.9) a	
3 min after reperfusion	91.2 (1.9) a,b	86.9 (1.9) a	
20 min after reperfusion	88.0 (1.7) a	84.8 (1.7) a,b	
60 min after reperfusion	87.8 (1.7) a	85.1 (1.7) a	
Surgery end	89.9 (1.7) a	84.9 (1.7) a	
**Cardiac index**			0.2340
After induction	3.7 (0.2)	3.3 (0.2)	
Preanhepatic	4.5 (0.2) a	3.9 (0.2) a	
Anhepatic	4.6 (0.2) a	3.8 (0.2)	
5 min before reperfusion	4.9 (0.2) a	3.9 (0.2) a	
3 min after reperfusion	5.2 (0.2) a,c	4.1 (0.2) a	
20 min after reperfusion	5.6 (0.2) a,b,c	4.8 (0.2) a,b	
60 min after reperfusion	5.7 (0.2) a,b	5.1 (0.2) a,b	
Surgery end	6.9 (0.7) a,b	4.8 (0.7)	
**Stroke volume index**			0.2774
After induction	47.2 (2.2)	43.6 (2.2)	
Preanhepatic	51.1 (1.6)	47.7 (1.7)	
Anhepatic	48.1 (1.9)	39.3 (2.0)	
5 min before reperfusion	53.0 (2.1) c	43.8 (2.1)	
3 min after reperfusion	56.0 (1.9) a,b,c	45.9 (1.9)	
20 min after reperfusion	62.4 (1.8) a,b,c	56.0 (1.8) a,b	
60 min after reperfusion	64.4 (2.0) a,b	58.7 (2.0) a,b	
Surgery end	61.4 (2.0) a,b	55.9 (2.0) a,b	
**Right ventricle end diastolic volume index**			0.1041
After induction	157.0 (6.1)	139.3 (6.6)	
Preanhepatic	143.4 (5.0) a	139.3 (5.2)	
Anhepatic	129.3 (4.6) a	109.5 (4.8) a	
5 min before reperfusion	135.5 (4.7) a	119.3 (4.9) a	
3 min after reperfusion	140.7 (4.7)	123.9 (4.9)	
20 min after reperfusion	160.7 (9.8) b	139.3 (10.1)	
60 min after reperfusion	148.8 (4.5) b	140.4 (4.6) b	
Surgery end	149.0 (4.1) b	141.4 (4.2) b	
**Systemic vascular resistance**			0.0939
After induction	1621.8 (128.3)	1764.7 (130.9)	
Preanhepatic	1287.2 (72.6) a	1632.8 (72.4)	
Anhepatic	1394.2 (89.8) c	1786.0 (89.5)	
5 min before reperfusion	1321.3 (91.7)	1704.2 (91.7)	
3 min after reperfusion	1134.0 (83.5) a,b	1411.2 (82.6) b	
20 min after reperfusion	875.0 (47.2) a,b	1095.3 (46.6) a,b	
60 min after reperfusion	880.3 (44.1) a,b	1043.7 (43.7) a,b	
Surgery end	850.6 (46.9) a,b,c	1136.2 (46.5) a,b	
**Mixed venous oxygen saturation**			0.3656
After induction	84.9 (1.1)	84.0 (1.2)	
Preanhepatic	87.3 (1.2) a	84.9 (1.2)	
Anhepatic	87.9 (0.9)	85.0 (0.9)	
5 min before reperfusion	103.0 (9.9)	86.4 (10.0)	
3 min after reperfusion	89.9 (0.7) a	88.6 (0.7) a	
20 min after reperfusion	87.8 (0.8) a	87.0 (0.8) a	
60 min after reperfusion	87.6 (1.0)	86.8 (1.0)	
Surgery end	87.2 (1.0)	85.9 (1.0)	

Data are presented as estimated mean (standard error).

TIVA, total intravenous anesthesia.

a Bonferroni-corrected *P* < 0.05 compared with the value of baseline (before induction or after induction) in each group.

b Bonferroni-corrected *P* < 0.05 compared with the value of 5 min before reperfusion in each group.

c Bonferroni-corrected *P* < 0.05 compared with the TIVA group.

### Intraoperative findings and postoperative outcomes

Surgery duration, hourly fluid infusion and urine output rates, transfused blood volume, and blood loss volume during surgery were similar in both groups (Table 4), as was the level of sufentanil consumption during anesthesia; however, intraoperative rates of vasopressor (norepinephrine and vasopressin) use were higher in the desflurane group than in the TIVA group. The duration of postoperative intensive care unit stay and overall hospitalization were similar between the groups. The incidence of reoperation or endoscopic intervention due to hepatic artery/portal vein thrombosis, bleeder ligation, hepaticojejunostomy site leak, bile duct leak or stricture were similar between the groups, as were graft failure and 6-month mortality rates. Postoperative cardiac complications occurred in seven patients, at a rate similar in both groups. Among these patients, five were in the desflurane group and had cardiac arrhythmias such as paroxysmal supraventricular tachycardia, or new-onset atrial fibrillation with rapid ventricular response; meanwhile, in the TIVA group, one patient experienced new-onset atrial fibrillation with rapid ventricular response and another experienced stress-induced cardiomyopathy. Intraoperative QTc values at several timepoints (baseline, after induction, and 5 min before and 3 min after reperfusion), rather than preoperative comorbidities or intraoperative hemodynamic factors, were risk factors for postoperative cardiac complications in univariable regression analysis (Table 5).

**Table 4:** Intraoperative data and postoperative outcomes.

	Desflurane group (n = 60)	TIVA group (n = 60)	*P* value
**Intraoperative data**			
Duration of surgery (mins)	553.5 (477.3, 614.5)	582.0 (522.8, 652.5)	0.131
Hourly fluid infusion (mL/kg/h)	13.3 (10.4, 16.8)	12.2 (9.9, 15.3)	0.401
Transfusion (mL)	1260.0 (600.0, 2399.0)	1080.0 (0.0, 2492.5)	0.243
Hourly urine output (mL/kg/h)	2.0 (1.2, 3.2)	2.1 (1.3, 3.3)	0.741
Intraoperative blood loss (mL)	4275.0 (2650.0, 6375.0)	4025.0 (2725.0, 7100.0)	0.935
Sufentanil (mcg/kg/h)	0.18 (0.15, 0.25)	0.20 (0.15, 0.27)	0.069
**Intraoperative vasopressor usage**			
Epinephrine (n, %)	11 (18.3)	7 (11.7)	0.306
Norepinephrine (mcg/kg/min)	0.05 (0.02, 0.13)	0.02 (0.01, 0.06)	0.001
Vasopressin (IU/h)	0.56 (0.10, 1.16)	0.13 (0.04, 0.45)	< 0.001
Intraoperative furosemide use (mg)	0.0 (0.0, 20.0)	0.0 (0.0, 10.0)	0.158
Intraoperative furosemide use (n, %)	29 (48.3)	19 (31.7)	0.062
Intensive care unit stay (day)	4.0 (3.0, 4.0)	4.0 (3.0, 4.0)	0.370
Hospital stay after surgery (day)	24.0 (19.0, 35.3)	23.0 (17.0, 37.8)	0.610
Postoperative cardiac complication (n, %)	5 (8.3)	2 (3.3)	0.439
Reoperation or endoscopic intervention within 3 months	12 (20.0)	11 (18.3)	> 0.999
Graft failure (n, %)	5 (8.3)	5 (8.3)	> 0.999
6 month mortality (n,%)	4 (6.7)	4 (6.7)	> 0.999

Data are presented as median (interquartile range) for continuous variables and count (percentage) for categorical variables.

TIVA, total intravenous anesthesia.

**Table 5:** Univariable logistic regression analysis of factors associated with postoperative cardiac complications.

variables	OR	95% CI	*P* value
**Preoperative factors**			
Female (vs. male)	1.541	0.328–7.241	0.584
ASA 4 (vs. ASA 3)	1.564	0.335–7.310	0.570
HTN	1.408	0.257–7.699	0.693
DM	1.221	0.261–5.720	0.800
Alcoholics (vs. nonalcoholics)	0.304	0.035–2.616	0.278
Hepatocellular carcinoma	5.492	0.640–47.084	0.120
Model for end-stage liver disease (MELD) score	1.040	0.949–1.140	0.397
QTc prolongation (vs. normal QTc)	1.012	0.187–5.488	0.989
**Intraoperative QTc**			
Baseline	1.023	1.002–1.044	0.028
After induction	1.015	1.001–1.029	0.039
5 min before reperfusion	1.018	1.006–1.030	0.004
3 min after reperfusion	1.013	1.003–1.023	0.011
20 min after reperfusion	1.008	0.998–1.017	0.119
60 min after reperfusion	1.010	1.000–1.019	0.053
Surgery end	1.007	0.998–1.015	0.132
**Hemodynamic factors 3 min after reperfusion**			
Cardiac index	1.120	0.789–1.589	0.527
Stroke volume index	1.011	0.964–1.060	0.6577
Right ventricle end-diastolic volume index	0.996	0.975–1.018	0.7158
Systemic vascular resistance index	0.999	0.998–1.001	0.3844
Mixed venous oxygen saturation (SvO_2_)	1.114	0.925–1.342	0.2559
**Postoperative laboratory data**			
TroponinT	0.986	0.957–1.016	0.361
NTproBNP	1.000	0.999–1.001	0.703
NGAL	0.996	0.977–1.016	0.717
procalcitonin	1.230	0.368–4.111	0.737

CI, confidence interval; OR, odds ratio; NGAL, neutrophil gelatinase-associated lipocalin; NTproBNP, N-terminal probrain natriuretic peptide.

Postoperative levels of troponin T and procalcitonin were higher than the corresponding baseline values measured 10 min after anesthetic induction. Changes to these parameters were similar in both groups (P_Group × Time_ = 0.535, and 0.341, respectively). Postoperative levels of N-terminal probrain natriuretic peptide (NTproBNP) and neutrophil gelatinase-associated lipocalin (NGAL) were comparable to baseline values. Hemoglobin levels were lower after than before surgery; changes to hemoglobin levels were similar in both groups (P_Group × Time_ = 0.180). While calcium levels decreased during surgery (P_Time_ < 0.001), potassium and magnesium levels remained unchanged, relative to the corresponding preoperative levels (P_Time_ = 0.064, and 0.150, respectively). Electrolyte changes during surgery were similar in both groups. The laboratory data are presented in the Supplementary Table S2.

## Discussion

In this study, the QTc interval was prolonged during liver transplantation in patients receiving both propofol-based TIVA and desflurane inhalational anesthesia; however, the former was associated with a diminished QTc prolongation compared to that linked with desflurane inhalational anesthesia. In patients with preoperative QTc prolongation relative to baseline values, further QTc prolongation was observed in the desflurane group but not in the TIVA group. Additionally, TIVA was associated with reduced vasopressor requirement during surgery, and may thus be a more suitable option than desflurane inhalational anesthesia for patients undergoing liver transplantation.

Compared to normal subjects, patients with cirrhosis have prolonged QTc^22,23^, and QTc prolongation occurs more frequently among patients with severe cirrhosis^24^. In particular, in end-stage liver disease, QTc prolongation is associated with mortality^25,26^; concurrently, in 75% of patients with prolonged QTc, QTc may normalize within a few weeks after liver transplantation^27^. However, during liver transplantation, QTc interval may prolong after anesthetic induction and remain prolonged throughout surgery^28,29^; the present study findings are consistent with those of previous studies. The mechanism of QTc prolongation during liver transplantation remains unclear; electrolyte imbalance, massive bleeding, hypotension, and consequent sympathetic hyperactivity may be involved^30–32^. Consequently, hypotension, hypocalcemia due to massive transfusion, and vasopressor use may underlie QTc prolongation in the present study population. Sympathetic stimulation due to endotracheal intubation may be another reason for QTc prolongation.

Since patients undergoing liver transplantation could be vulnerable to QTc prolongation, the choice of anesthetic agents is important for this population. Except for halothane, most of the commonly used inhalational agents, such as isoflurane, sevoflurane, and desflurane are reported to prolong the QTc^12,14,16,17,33,34^. In a study on liver transplantation, both sevoflurane and desflurane increased the QTc, and there was no difference in QTc values between the two agents^29^. In contrast, the effect of propofol on the QTc is not consistent but seems more favorable than inhalational agents. Most of the previous studies demonstrated that propofol does not prolong the QTc^16,17,34^, and even shortens it^19,35^. However, some investigators have reported QTc prolongation by propofol^20,21^.

To our knowledge, this study is the first to compare the effect of inhalational anesthesia and propofol-based TIVA on QTc interval during liver transplantation. Changes to QTc observed during surgery differed between the groups. The pungency of desflurane can irritate the airway, leading to sympathetic stimulation during anesthetic induction^15^. Desflurane may also increase plasma renin activity, and plasma levels of catecholamines such as epinephrine and norepinephrine^36^. In the present study, cardiac index and stroke volume index values increased in both groups; in addition, after reperfusion, they were higher in the desflurane group than in the TIVA group. Hemodynamic instability due to abrupt potassium increase, exacerbation of systemic vascular resistance, and myocardial depression in postreperfusion period are well-documented^32,37^, and ventricular arrhythmias, including torsades de pointes, in peri-reperfusion period have been previously reported^4–6^. Although propofol-based TIVA did not totally offset QTc prolongation during liver transplantation, given the susceptibility to life-threatening arrhythmias of patients with severely prolonged QTc of > 500 ms^38^, the observed preventive effect of propofol on QTc prolongation during surgery may be meaningful. Furthermore, in patients with preoperative QTc prolongation, QTc remained stable throughout surgery in the TIVA group; however, the underlying mechanism is unclear. A previous study has reported the shortening of QTc after anesthetic induction using propofol in patients with baseline QTc of > 440 ms, but not in patients with baseline QTc of < 440 ms^39^.

Previous studies on the effect of liver transplantation on QTc changes have focused on the preoperative and postoperative values of QTc^40–43^, and only a few assessed intraoperative QTc changes^28,29^. In addition, previous studies have reported the normalization or improvement of QTc values several months after liver transplantation; however, in the present study, postoperative QTc findings normalized to preoperative values on postoperative day 3. Overall, the present findings suggest a protective effect of propofol on QTc prolongation during surgery and until postoperative day 1, specifically, in patients with preoperative QT prolongation. No similar effect was observed in the desflurane group.

It should be noted that the TIVA group had lower vasopressor needs during surgery than did the desflurane group. The cardioprotective effect of propofol against ischemia reperfusion injury may help maintain hemodynamic stability. In a previous study, propofol infusion during liver transplantation helped attenuate the formation of lipid peroxide, suggesting an antioxidant effect of propofol^44^. Propofol has been shown to exert cardioprotective effects during ischemia reperfusion injury via various pathways including antioxidant, free radical scavenging activities, and anti-inflammatory responses^45–48^.

This study has some limitations. First, many patients included were receiving medications such as calcium channel blockers or beta blockers, which may affect the QT interval during surgery^30^. However, these medications are commonly used in liver transplantation recipients, and there was no difference in medication types between the two groups in this study (Table 1). Second, the dose of vasopressors used during surgery differed between the groups; vasopressors influence sympathetic activation, which is among the causes of QTc prolongation. However, this observed difference may be due to the antioxidative and cardioprotective effects of propofol^44–48^, which is likely representative of the clinical picture. Third, while data on intraoperative QTc values were collected using LabChart software, the preoperative and postoperative QTc findings were assessed using 12-lead electrocardiogram (ECG) in the ward or intensive care unit. The use of different measuring methods may have affected the presented estimates; thus, we additionally compared postoperative QTc findings with preoperative values. Fourth, propofol was used for anesthetic induction for patients in the desflurane group. However, most intravenous agents, such as thiopental, etomidate, and ketamine, are also known to prolong the QTc. Since propofol may have the effect of reducing QTc prolongation, its use for induction helps to further reveal the difference between desflurane and propofol in our results. Lastly, given that the analysis of postoperative complications was outside the scope of the present study, the sample size does not have enough power to detect a clinically relevant difference. Further studies are required to determine the effect of these anesthetic methods on postoperative outcomes.

In conclusion, this study shows that propofol-based TIVA may reduce QTc prolongation during living donor liver transplantation compared with that observed under desflurane inhalational anesthesia. The protective effect of TIVA against QTc prolongation was more favorable in patients with preoperative QTc prolongation than in their counterparts. Furthermore, liver transplantation under TIVA required less intraoperative vasopressor use than that under inhalation-based anesthesia.

## Methods

The present study protocol was approved by the Institutional Review Board of the Yonsei University Health System, Seoul, South Korea (#4-2018-1164) on 15 February 2019 and registered at ClinicalTrials.gov (NCT03864276; 06/03/2019). This study was conducted in accordance with the ethical guidelines of the Helsinki Declaration. We received written informed consent from all patients enrolled in this study, which was conducted from March 2019 to December 2020.

This study included 120 patients aged ≥ 20 years with American Society of Anesthesiologists physical status class III-IV, who underwent living donor liver transplantation. Patients with unstable angina, recent myocardial infarction, uncontrolled hypertension, implantable cardiac defibrillator, obesity (body mass index of > 30 kg/m^2^), and allergy to propofol were excluded. All patients were randomly allocated to either the TIVA or desflurane group at a 1:1 ratio, using a computer-generated random code generator. Investigators and patients were blinded to group assignment, except for the attending anesthesiologists. Surgeries were performed by one of two transplantation surgeons.

### Anesthetic management and study design

Standard monitoring, including pulse oximetry, non-invasive blood pressure monitoring, and ECG, was performed for all patients. In the desflurane group, 2 mg/kg propofol was used for induction, and general anesthesia was maintained with 3–7% desflurane. In the TIVA group, propofol was used to induce and maintain general anesthesia by an effect-site target-controlled infusion pump (Orchestra Base Primea: Fresenium Vial, Brezins, France), according to the Marsh model. In all patients, 0.1 mg/kg sufentanil and 0.6 mg/kg rocuronium were used before endotracheal intubation, and these agents were manually infused throughout the surgery. Depth of anesthesia was maintained using a Sedline electroencephalogram sensor (Masimo Corp., Irvine, CA, USA) at target patient state index range between 25 and 50.

Right radial and femoral arteries were cannulated, and a pulmonary artery catheter (Swan-Ganz CCOmbo, Edwards Lifescience) was inserted in the internal jugular vein through the 9-Fr large-bore catheter (Advanced Venous Access Device, Edwards Lifesciences, Irvine, Calif, United States).

Norepinephrine was infused to treat hypotension with a mean femoral arterial pressure lower than 65 mmHg. When the cardiac index was higher than 2.5 L/min/m^2^ and systemic vascular resistance index was lower than 2000 dynes · sec/cm^5^/m^2^, vasopressin infusion was added to treat hypotension.

### Data collection

The primary outcome was the impact of the two anesthetic methods on QTc changes during living donor liver transplantation. Secondary outcomes were perioperative changes to the Tp-e interval and Tp-e/QT ratio. ECG data, including QTc and Tp-e interval values, were averaged from four consecutive beats and collected serially using LabChart software (Pro version 7; AD Instruments, Colorado Springs, CO) and a data acquisition system (PowerLab; AD Instruments) at the following timepoints: baseline (before induction); after induction (30 min after endotracheal intubation); preanhepatic phase; anhepatic phase; 5 min before reperfusion; 3, 20, and 60 min after reperfusion, and at the end of surgery.

In addition, QTc values based on 12-lead ECG data were collected preoperatively, and on postoperative days 1, 2, and 3. Vital signs (mean blood pressure, heart rate) were collected at the same timepoints as the ECG data. Other hemodynamic data including cardiac index, stroke volume index, right ventricle end diastolic volume index, systemic vascular resistance, mixed venous oxygen saturation values and body temperature were collected via a pulmonary artery catheter at the following timepoints: after induction (after the swan ganz catheter insertion); preanhepatic phase; anhepatic phase; 5 min before reperfusion; 3, 20, and 60 min after reperfusion, and at the end of surgery.

Data on perioperative characteristics, including duration of surgery; fluid intake volume; transfusion volume; urine output; blood loss volume; sufentanil consumption volume; vasopressor usage; diuretics (furosemide) usage; intensive care unit stay duration; hospitalization duration; postoperative cardiac complication rates, including new-onset atrial fibrillation, paroxysmal supraventricular tachycardia, and stress-induced cardiomyopathy; reoperation or endoscopic intervention due to any reasons related to surgical complications within 3 months after the transplantation and graft failure; 6-month mortality rates, and laboratory findings (levels of troponin T, NTproBNP, NGAL, and procalcitonin) were collected. The primary and secondary outcomes were compared between the groups (desfurane vs. TIVA group).

For secondary analyses, the patients were classified into two subgroups: preoperative normal QTc group, and preoperative QTc prolongation group. Patients with the QTc of > 450 ms or > 470 ms in men and women, respectively, confirmed by preoperative ECG findings, were classified as presenting with preoperative QTc prolongation. The impact of anesthetics on QTc changes was examined in these subgroups.

### Statistical analyses

Previous studies have reported the mean value (standard deviation) of the corrected QT interval after reperfusion in patients undergoing living donor liver transplantation under inhalational anesthesia as 452 ± 55 ms^29^. We considered a 30-ms reduction of the QTc as clinically relevant. Given a dropout rate of 10%, 120 patients were required for analyses with 80% power at a significance level of 0.05.

Descriptive variables were presented as median (interquartile range) or estimated mean (standard error) for continuous variables, and as counts (percentage) for categorical variables. For between-group comparisons, Fisher’s exact tests and independent *t*-tests were used for categorical variables and continuous variables, respectively. We computed linear mixed models with group, time, and interaction between group and time for repeated variables (ECG data [QTc interval, Tp-e interval, Tp-e/QT ratio], mean blood pressure, heart rate, cardiac index, stroke volume index, right ventricle end diastolic volume index, systemic vascular resistance value, and mixed venous oxygen saturation levels). Post-hoc analysis was performed using the Bonferroni correction for within- and between-group comparisons. Factors associated with postoperative cardiac complications were determined by univariable regression analysis, which included factors such as preoperative comorbidities, intraoperative QTc values, hemodynamic variables, and laboratory findings. Statistical analyses were performed using SPSS Statistics for Windows (version 25; IBM Corp., Armonk, NY, USA), SAS (version 9.4, SAS Inc., Cary, NC, USA), R package, version 3.6.0 (The R Foundation for Statistical Computing, Vienna, Austria), or PASS (version 12, NCSS, LLC, Kaysville, Utah, USA).

## Supplementary Information

Supplementary Table S1.

Supplementary Table S2.

## Data Availability

The raw data of this study are available from the corresponding author on request.
