# Supplementary material for: Propofol prevents further prolongation of QT interval during liver transplantation
Source: Sci Rep. 2022 Mar 17;12:4636. doi: 10.1038/s41598-022-08592-4 (PMC8931121; doi:10.1038/s41598-022-08592-4)
Supplement: Supplementary file 1 — Supplementary Table S1. [file 41598_2022_8592_MOESM1_ESM.docx]

Table S1. Changes in body temperature (°C) during surgery

|  | Desflurane group (n= 60) | TIVA group (n=60) | P value |
| --- | --- | --- | --- |
| After induction | 35.7 (35.4, 36.0) | 35.9 (35.4, 36.1) | 0.179 |
| Preanhepatic | 35.8 (35.4, 36.1) | 35.8 (35.4, 36.2) | 0.848 |
| Anhepatic | 36.2 (35.7, 36.5) | 36.2 (35.8, 36.7) | 0.333 |
| 5 mins before reperfusion | 35.9 (35.7, 36.3) | 36.0 (35.8, 36.4) | 0.850 |
| 3 mins after reperfusion | 35.8 (35.5, 36.1) | 35.9 (35.5, 36.2) | 0.269 |
| 20 mins after reperfusion | 35.9 (35.6, 36.2) | 36.0 (35.5, 36.4) | 0.244 |
| 60 mins after reperfusion | 36.0 (35.6, 36.3) | 36.1 (35.6, 36.5) | 0.724 |
| Surgery end | 36.5 (36.1, 36.8) | 36.5 (36.1, 6.9) | 0.351 |

Data are presented as median (interquartile range).
