# Supplementary material for: Propofol prevents further prolongation of QT interval during liver transplantation
Source: Sci Rep. 2022 Mar 17;12:4636. doi: 10.1038/s41598-022-08592-4 (PMC8931121; doi:10.1038/s41598-022-08592-4)
Supplement: Supplementary file 2 — Supplementary Table S2. [file 41598_2022_8592_MOESM2_ESM.docx]

Table S2. Changes in laboratory data during surgery

|  | Desflurane group (n= 60) | TIVA group (n=60) | P_time*group_ |
| --- | --- | --- | --- |
| TroponinT (pg/mL) |  |  | 0.535 |
| Preoperative | 9.0 (6.0, 13.0) | 8.0 (6.0, 15.8) |  |
| postoperative | 29.0 (20.0, 65.0) | 37.0 (18.3, 58.8) |  |
| NTproBNP (pg/mL) |  |  | 0.180 |
| Preoperative | 133.0 (67.5, 363.0) | 82.0 (41.5, 218.5) |  |
| postoperative | 209.0 (115.3, 423.8) | 133.0 (80.0, 348.0) |  |
| NGAL (ng/mL) |  |  | 0.886 |
| Preoperative | 70.7 (52.7, 110.6) | 66.6 (54.8, 90.3) |  |
| postoperative | 86.6 (67.7, 119.7) | 85.5, (70.4, 117.1) |  |
| Procalcitonin (ng/mL) |  |  | 0.341 |
| Preoperative | 0.09 (0.05, 0.15) | 0.06 (0.04, 0.17) |  |
| postoperative | 0.22 (0.13, 0.62) | 0.23 (0.11, 0.47) |  |
| Hemoglobin (g/dL) |  |  | 0.180 |
| Preoperative | 10.5 (8.9, 12.1) | 11.5 (9.1, 13.2) |  |
| postoperative | 8.4 (7.6, 9.1) | 8.6 (8.0, 9.6) |  |
| Potassium (mmol/L) |  |  | 0.357 |
| Before induction | 4.1 (3.8, 4.3) | 4.1 (3.8, 4.4) |  |
| After induction | 3.5 (3.2, 3.9) | 3.5 (3.1, 3.8) |  |
| Anhepatic | 3.6 (3.3, 4.0) | 3.5 (3.2, 3.9) |  |
| 20 mins after reperfusion | 3.6 (3.3, 4.0) | 3.7 (3.3, 3.9) |  |
| Surgery end | 3.8 (3.5, 4.1) | 3.7 (3.5, 4.0) |  |
| Calcium (mg/dL) |  |  | 0.420 |
| Before induction | 4.9 (4.7, 5.0) | 4.8 (4.7, 4.9) |  |
| After induction | 4.6 (4.4, 4.8) | 4.6 (4.4, 4.8) |  |
| Anhepatic | 4.7 (4.5, 4.9) | 4.7 (4.5, 4.9) |  |
| 20 mins after reperfusion | 4.8 (4.5, 5.0) | 4.7 (4.5, 5.0) |  |
| Surgery end | 4.9 (4.6, 5.2) | 5.0 (4.8, 5.1) |  |
| Magnesium (mg/dL) |  |  | 0.324 |
| Before induction | 1.5 (1.3, 1.8) | 1.6 (1.3, 1.7) |  |
| After induction | 1.3 (1.2, 1.5) | 1.4 (1.2, 1.5) |  |
| Anhepatic | 1.4 (1.2, 1.5) | 1.4 (1.3, 1.6) |  |
| 20 mins after reperfusion | 1.3 (1.2, 1.5) | 1.4 (1.3, 1.5) |  |
| Surgery end | 1.3 (1.2, 1.6) | 1.4 (1.3, 1.5) |  |

NGAL, neutrophil gelatinase-associated lipocalin; NTproBNP, N-terminal probrain natriuretic peptide.

Data are presented as median (interquartile range).
